# A Rare Case of Paraneoplastic Syndrome Presented with Severe Gastroparesis due to Ganglional Loss

**DOI:** 10.1155/2012/894837

**Published:** 2012-12-04

**Authors:** Konstantinos N. Argyriou, Martin Peters, Javaid Ishtiaq, Santosh Enaganti

**Affiliations:** ^1^Gastroenterology Department, Diana, Princess of Wales Hospital, South Humberside DN33 2BA, UK; ^2^Histopathology Department, Diana, Princess of Wales Hospital, South Humberside DN33 2BA, UK

## Abstract

Paraneoplastic syndromes are rare initial manifestations of a neoplastic disorder that may precede the actual detection of an overt cancer. These syndromes can generally involve any organic system of the human body with gastroparesis being the commonest manifestation of the paraneoplastic involvement of the neuronal bodies of the gastrointestinal tract in cancer patients. Gastroparesis is the result of an autoimmune destruction of the nerve plexus of the stomach that causes nonspecific gastrointestinal symptoms such as intractable vomiting and abdominal discomfort that interfere with patients' quality of life and are often ascribed to psychological factors. Thus, if not suspected, it easily evades the diagnostic thought especially in those cases where the diagnostic work up has not detected any apparent cause. Consequently, it should always be considered in patients with diagnosed or suspected cancer who complain of unexplained gastrointestinal symptoms. In our report, so as to increase the clinical awareness of this rare clinical entity, we present the case of a 70-year-old Caucasian female who presented in our hospital with severe gastroparesis that was later proven to be associated with an overt small cell lung cancer (SCLC) and we discuss the existing knowledge of the pathophysiology, diagnosis, and management of this disorder.

## 1. Introduction

Paraneoplastic syndromes represent a group of nonmetastatic systemic disorders that accompany malignant disease and are usually the first or most prominent manifestation of an underlying malignancy [1].

Generally, paraneoplastic syndromes occur in 7–15% of cancer patients and can involve all the organic systems of the human body with different frequencies [2]. Neurological involvement is found in only 0.01% of cancer patients and can be sensory, motor, mixed somatic, or autonomic leading to various syndromes and disorders [3].

Gastroparesis is the commonest manifestation of the paraneoplastic involvement of the neuronal bodies of the gastrointestinal tract in cancer patients. It affects the stomach and is characterized by delayed gastric emptying in the absence of mechanical obstruction with its pathophysiology to remain mostly unknown. However, most studies suggest an autoimmune destruction of the neuronal (also called mesenteric) plexus of the stomach as the most prevalent mechanism for its development [4]. It presents with nonspecific gastrointestinal symptoms such as postprandial dyspepsia, early satiety, nausea/vomiting, and bloating and its diagnosis requires high clinical suspicion especially in those cases where its presentation precedes the diagnosis of the primary tumor [4, 5].

In order to increase clinical awareness on this rare clinical entity, we present a case of this interesting disorder and we quote the existing knowledge on its diagnosis and management.

## 2. Case Report

In November 2011, a 70-year-old Caucasian female with a background of essential hypertension, diverticulosis, and mild chronic obstructive pulmonary disease presented with an eight week history of significant weight loss (24 kilos), early satiety, postprandial dyspepsia, and nausea with intractable vomiting right after recovering from a lower respiratory tract infection for which she completed two courses of antibiotics in the community (amoxicillin and clarithromycin) with complete recovery. She was a life long smoker of 10–20 cigarettes per day and was drinking thirty units of alcohol weekly. She had no family history of gastric or bowel malignancy.

On clinical examination, her body mass index was reduced with no pallor or jaundice. Respiratory examination revealed slightly decreased air entry bilaterally but otherwise was unremarkable.

Routine electrocardiogram showed sinus rhythm with occasional premature ectopics. Her relevant laboratory findings including liver function tests, urea and electrolytes, bone profile, amylase, C-reactive protein, tumor markers, and full blood count were all within the normal range, except for a low number of platelets and prolonged aPTT.

Initial differential diagnosis included gastric outlet obstruction, malignancy, and peptic ulceration. Chest and abdominal X-ray ruled out mechanical obstruction. Upper and lower gastrointestinal tract endoscopy revealed erythematous mucosa in the body of the stomach and multiple sigmoid diverticulae but ruled out ulceration, obstruction, and malignancy.

Further investigation with abdominal-pelvic computed tomography and magnetic resonance imaging of her small bowel showed few clinically insignificant incidental non-gastrointestinal abnormalities but no other evidence of intra-abdominal pathology.

Having ruled out mechanical obstruction and in view of persistence of intractable vomiting, gastroparesis was suspected clinically even though there were no findings suggestive of gastric stasis or dilatation in the previous investigations. Thus, patient was further investigated with gastric emptying studies which was found to be grossly abnormal with half emptying time to be approximately three times the upper normal limit for patient's age, confirming a diagnosis of severe gastroparesis.

Initially, patient was conservatively treated with the combination of metoclopramide-erythromycin and dietary modifications for small frequent low in fat semisolid meals but, due to inadequate control of her symptoms and further deterioration of her nutritional status, it was decided that nasojejunal feeding followed by total parenteral nutrition was required. Her clinical condition improved and patient was then referred for consideration of temporary and if well tolerated permanent gastric pacing.

Following the insertion of the gastroscopic neuromodulator wires and despite the improvement of patient's symptom's score, gastric emptying study further deteriorated and permanent gastric pacing was deferred based on current evidence.

As nasojejunal feeding was well tolerated and long term total parenteral nutrition was declined by the patient, distal gastrectomy with a wide Roux en Y gastrojejunostomy was considered in order not only to facilitate gravity led gastric emptying but also to prevent aspiration.

Patient underwent the procedure uneventfully and the excised specimen of the stomach was found to be largely devoid of ganglion cells which extended up to the resection margins in the duodenum with associated chronically inflamed nerve fibers suggestive of gastroparesis due to nerve injury (Figure 2).

Postoperatively, although intractable vomiting and nausea improved, patient could only tolerate small amount of enteral nutrition which was inadequate to meet her nutritional needs.

One week later, patient developed severe nosocomial lower respiratory tract infection with *Klebsiella pneumoniae* and *Pseudomonas aeruginosa* requiring intensive care unit admission and, unfortunately, died three weeks postoperatively.

Premortem CT scans and bronchoscopy did not show signs of malignancy but postmortem examination revealed evidence of small cell lung carcinoma (SCLC) with malignant cells to be present along the excised thoracic lymph nodes but without identification of the primary tumor.

## 3. Discussion

In the majority of cases, paraneoplastic syndromes are usually the first or most prominent manifestation of an underlying malignancy. But, in some cases they can precede tumor diagnosis with the mean latency to range between two and twelve months [1].

Although paraneoplastic disorders can involve any organic system of the human body, the involvement of the neurological system is extremely rare [3, 6, 7].

Among different types of malignancy, lung cancer, SCLC in particular, is the type which is more often associated with the development of a paraneoplastic disorder of the nervous system [3, 6, 7].

In SCLC, neurologic syndromes include the Lambert-Eaton myasthenic syndrome, limbic encephalopathy, polyneuropathy), cerebellar degeneration, retinopathy, opsoclonus-myoclonus, and autonomic neuropathy with autoimmune mechanisms to play a crucial role in their development through autoantibodies that directs against ligand- or voltage-gated channels to cause changes in synaptic function or neuronal excitability [8–10]. Among the different types of autoantibodies, anti-Hu autoantibodies have been identified to play a crucial role in the pathogenesis of paraneoplastic neuropathies and represent a useful diagnostic marker in the early diagnosis of these disorders since they recognize a family of RNA-binding proteins (HuD, HuC, Hel-N1, and Hel-N2) expressed in the nuclei of neurons and cancer cells which are initially driven to control tumor growth but later misdirected and cause the neurological damage [10].

Clinically, paraneoplastic neuropathies present with features that correspond to the underlying neurological damage with various motor and sensory symptoms and signs which become absent in cases where the autonomic nervous system is involved [4, 11].

In autonomic neuropathy, patients present with hypothermia, hypoventilation, sleep apnoea, intestinal pseudo-obstruction, gastroparesis, and cardiac arrhythmias with gastroparesis to be present in up to 60% of those who have gastrointestinal symptoms and suffer from cancer [12].

Gastroparesis is the disorder of the stomach which is characterized by delayed gastric emptying in absence of mechanical obstruction and should be suspected in patients with known or unknown cancer with nonspecific gastrointestinal symptoms such as nausea/vomiting, early satiety and bloating, and signs of autonomic neuropathy when mechanical obstruction and other common gastrointestinal disorders have been excluded [4].

**Figure 1:**
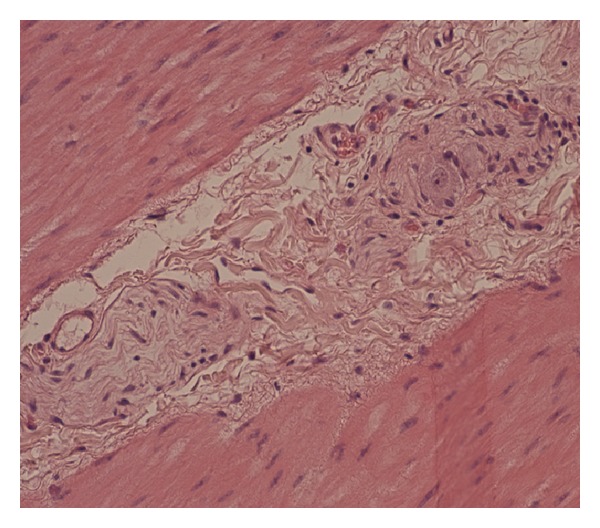
Intermesenteric plexus of a normal stomach. Normal nerve and a ganglion cell. (Haematoxylin-Eosin, ×200).

**Figure 2:**
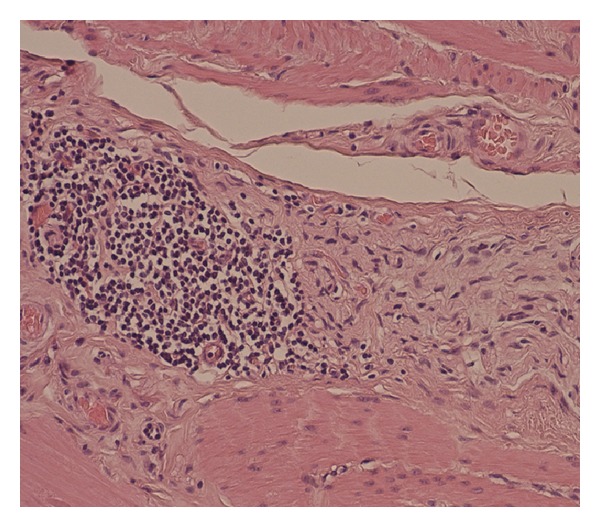
Intermesenteric plexus of the abnormal stomach. Nerve infiltrated by lymphocytes and devoid of ganglion cells. (Haematoxylin-Eosin, ×200).

Although the exact pathophysiological mechanism of gastroparesis remains unknown, at autopsy of patients with paraneoplastic gastroparesis such as in our case, the stomach has been found to be largely devoid of neuronal ganglions with chronically inflamed nerve fibers (Figures 1 and 2) which together with the serological detection of anti-Hu autoantibodies suggest an autoimmune destruction of the neuronal plexus of the stomach as the main mechanism for its development [4, 13, 14].

Diagnostically, in patients with known non-gastrointestinal cancer, mechanical obstruction and other common gastrointestinal disorders should be initially excluded with the combination of upper and lower gastrointestinal endoscopy or gastrointestinal follow through and radiological imaging with X-ray, computed tomography and magnetic resonance. Then, patients should undergo gastric emptying studies so as to confirm the diagnosis [4].

But, in overt cancer and as full thickness biopsy of the stomach is not a routine practice, current evidence suggests that serological detection of anti-Hu antibodies are of clinical importance in the early diagnosis of paraneoplastic gastroparesis with a specificity and a sensitivity that reaches 99% and 82%, respectively [15]. However, in our case, anti-Hu autoantibodies could not be determined due to lab related issues (blood sample was sent from our immunology laboratory to the laboratory of Scunthorpe General Hospital for processing but lost without early notification of the authors so as to resend a new one).

Once the diagnosis is established the treatment is mainly supportive, the prognosis is poor, and our therapeutic targets are symptoms' relief and improvement of patient's health related quality of life [4, 16, 17].

In general, most studies suggest two complementary approaches for the management of paraneoplastic gastroparesis basing on its immune-mediated hypothesis: removal of the antigen source by treating the tumour and suppression of the immune response. But therapeutic interventions such as diet modifications, pharmacological agents, therapeutic endoscopy, surgery, and psychological counseling are also needed since they contribute to a better outcome [4, 16, 17].

Dietary modifications have their own role in the management of paraneoplastic gastroparesis but the expected clinical benefit is often modest. Fat delays emptying and nondigestible fibre (e.g., fresh fruits and vegetables) may be poorly emptied. Thus, patients should be advised to consume a low-fat diet (without nondigestible fibre) and frequent, small meals under the assistance and education of a dietician. In more severe cases, as in our case, substitution of mixed meals with homogenized or liquid meals supplemented with vitamins may be helpful but if symptoms persist and the metabolic panel of the patient deteriorates, enteral nutrition via a jejunostomy tube is required. Parenteral nutrition should be restricted to patients with severe gastric and small intestine dysmotility in whom enteral feeding becomes impossible [4, 16, 17].

Prokinetic agents (motilin receptor agonists, serotonin 5-HT4 receptor agonists, cisapride, dopamine antagonists), steroids (dexamethasone), and antiemetics (serotonin 5-HT3 receptor antagonists, tricyclic antidepressants, phenothiazines) have been all been used in the management of paraneoplastic gastroparesis with their use resulting in subjective improvement in symptoms of nausea, vomiting, abdominal pain, postprandial fullness, nausea, and early satiety in patients with advanced cancer. However, in our case, their effectiveness was less than ideal since our patient's mesenteric plexus was largely devoid of ganglions with severe inflammatory changes of its nerve fibers [4, 16, 17].

Other than pharmacologic interventions, in refractory cases of paraneoplastic gastroparesis, various non pharmacologic interventions have been used in order to provide access for enteral nutrition, as well as gastric decompression, and thereby palliation [4, 16, 17].

Generally, the enteral route is preferable over the parenteral route for feeding even in severe cases of gastroparesis since this approach prevents patients from complications that increase morbidity and subsequent hospitalization such as venous thrombosis, line sepsis, and volume overload which are aligned with the parenteral nutrition [4, 16, 17].

In order to decompress the hypotonic and static stomach or gut and provide access for enteral nutrition, there have been various major and minor surgical interventions with different outcomes tried [4, 16, 17].

Major gastric resections such as partial or total gastrectomy with Roux-en-Y reconstruction have been carried out in patients with paraneoplastic gastroparesis with different outcomes. But, since cancer patients are usually medically compromised, the benefits of providing a route for artificial nutrition should be always weighed against the risks of the procedure itself and the prognosis of the underlying malignancy [4, 16, 17].

Alternatively to major resections, percutaneous, laparoscopic, or transpyloric inserted tubes for feeding represent minor surgical interventions that have been also tried in patients with severe gastroparesis. However, these interventions are also associated with potentially serious complications such as aspiration, blockage, easy displacement, and infections and their selection should be made meticulously [4, 16, 17].

In contrast, gastric electrical stimulation represents a novel therapeutic option that may be of use in the management of malignant gastroparesis without the need of a major surgery. This intervention involves endoscopic or surgical placement of a pacemaker-like device that delivers high frequency, low-amplitude stimulation via electrodes implanted in the gastric muscle. But, due to its limited use and the absence of long term controlled studies, routine use of gastric neuromodulators in patients with gastroparesis who are unfit for surgery cannot be recommended [4, 16, 17].

In conclusion, after ruling out common aetiologies first, gastroparesis should always be considered in patients with diagnosed or suspected cancer who complain of nonspecific gastrointestinal symptoms, with serological autoantibodies (anti-Hu) and full thickness biopsy of the stomach, to be of specific importance for its diagnosis. Its treatment is mainly supportive and involves pharmacological, non-pharmacological and dietary measures with different outcomes. No recommendations can be made for the long term management plan of the cancer patients who suffer from paraneoplastic gastroparesis since there is absence of relevant controlled studies with further studies required on this topic.
